# Visual body size norms and the under‐detection of overweight and obesity

**DOI:** 10.1002/osp4.143

**Published:** 2017-12-21

**Authors:** M. Oldham, E. Robinson

**Affiliations:** ^1^ Psychological Sciences University of Liverpool Liverpool UK

**Keywords:** Body size norms, obesity, visual perception, weight misperceptions

## Abstract

**Objectives:**

The weight status of men with overweight and obesity tends to be visually underestimated, but visual recognition of female overweight and obesity has not been formally examined. The aims of the present studies were to test whether people can accurately recognize both male and female overweight and obesity and to examine a visual norm‐based explanation for why weight status is underestimated.

**Methods:**

The present studies examine whether both male and female overweight and obesity are visually underestimated (Study 1), whether body size norms predict when underestimation of weight status occurs (Study 2) and whether visual exposure to heavier body weights adjusts visual body size norms and results in underestimation of weight status (Study 3).

**Results:**

The weight status of men and women with overweight and obesity was consistently visually underestimated (Study 1). Body size norms predicted underestimation of weight status (Study 2) and in part explained why visual exposure to heavier body weights caused underestimation of overweight (Study 3).

**Conclusions:**

The under‐detection of overweight and obesity may have been in part caused by exposure to larger body sizes resulting in an upwards shift in the range of body sizes that are perceived as being visually ‘normal’.

## Introduction

Although the worldwide prevalence of obesity has increased dramatically over the last 30 years 1, there is evidence suggesting that overweight and obesity often go undetected. Individuals with overweight and obesity consistently underestimate their own weight status 2. Furthermore, a number of studies show that both parents 3, 4 and healthcare professionals (HCPs) 5, 6 visually underestimate the weight status of children and patients with overweight and obesity. One potential explanation is that increases in the prevalence of obesity may have resulted in an upwards shift in the range of body sizes that are perceived visually as being ‘normal’ and that this may have resulted in widespread under‐detection of overweight and obesity.

Perceptions of stimulus normality form a critical point of reference when making visual judgements 7, 8, 9. In relation to body size, this type of ‘norm comparison’ process would predict that when judging whether a target body is overweight or not, the target body is compared with a person's internal visual perception of a ‘normal’ body size, and target bodies will only be judged as being overweight if they appear larger than the ‘norm’. In support of this theory, a large‐scale study found that accurate perception of overweight was more likely as a person's body size moved away from the population average or ‘normal’ body size 10. The exact type of ‘norm comparison’ that is made when judging weight status is less clear. One proposal is that when making visual judgements, stimuli are compared against an internal norm or ‘prototype’ of what is perceived as being the average size 9. Another explanation is that body size is perceived categorically 11. Thus, for each observer, there may be a particular range of body sizes that are perceived as normal, and it is only when a person's body size is above the upper boundary of this ‘norm range’ that they are perceived as being overweight.

If weight status is judged according to a ‘norm comparison’ process 12, frequent exposure to heavier body weights could contribute to under‐detection of overweight and obesity by recalibrating perceptions of what constitutes a ‘normal’ body size. This is because visual body size norms are likely to be based on the size of bodies that are frequently seen in the environment, otherwise known as the ‘visual diet’ 7, 9, 13. Cross‐sectional data suggest that underestimation of personal weight status is more common when there is a high prevalence of obesity in the local area 14, 15. There is also experimental evidence indicating that visual exposure to heavier bodies may increase underestimation of weight status 16, result in greater visual preference for larger bodies 17 and increase the body sizes, which are perceived as being ‘normal’ 18. Therefore, increases in obesity prevalence may have shifted the range of body sizes that appear ‘normal’ and, in turn, impacted the visual recognition of obesity.

A previous study has shown that absolute body weight (e.g. in kgs) tends to be visually underestimated 19, but there has been little direct experimental testing of whether the lay public can objectively visually identify overweight and obesity. Some work has suggested that male overweight and obesity tends to be visually underestimated 6, 20. However, there has been no systematic examination of visual identification of female overweight and obesity. This is of importance because there may be sex differences in the visual identification of male and female overweight and obesity. For example, parents 21, 22 and HCPs 23, 24 are more likely to underestimate overweight and obesity when a person is male, as opposed to female. Furthermore, there are different cultural body ideals for men and women, and more emphasis is placed on the value of thinness for women 25. As such, perceptions of the normal female body may be smaller than the normal male body, which could result in more accurate identification of female overweight and obesity.

This manuscript examines whether exposure to obesity has led to an upwards shift in terms of what is considered a normal body size and whether this results in greater visual underestimation of overweight and obesity. No studies have examined whether visual body size norms explain when overweight and obesity go visually under‐detected. Three experimental studies are reported. Study 1 examined whether the weight status of men and women with normal weight, overweight and obese body mass index (BMI) was visually underestimated. Study 2 investigated whether body size norms explained underestimation of overweight and obesity. Finally, Study 3 examined whether visual exposure to obesity alters body size norms and whether this process leads to underestimation of overweight body sizes. The first hypothesis was that participants would frequently underestimate the weight status of men and women with overweight and obesity but that the level of underestimation may be less pronounced for female, as opposed to male, overweight and obesity (Study 1). The second hypothesis was that body size norms would predict underestimation, whereby those who thought larger bodies were more normal would be most likely to underestimate overweight and obesity (Study 2). The third hypothesis was that exposure to heavier bodies would lead to larger body size norms, and this would in turn increase the likelihood of overweight body sizes being visually underestimated (Study 3).

## Study 1

Study 1 was designed to examine whether members of the general public are able to accurately identify the weight status of men and women with normal weight, overweight and obese BMIs.

### Methods

#### Participants

One hundred and three US participants were recruited via Amazon Mechanical Turk (MTurk), which has been identified as a valid online recruitment method 26, 27. For example, Casler and colleagues found that data collected using Amazon MTurk was equivalent to data collected in a laboratory study and that MTurk offered a more varied sample than a traditional laboratory approach 26. Data quality was ensured by only recruiting MTurk participants with a previous approval rating of ≥95% 28. Participants were asked to complete the survey on a computer or laptop to avoid distortion to images, and the device used was recorded at the end of the survey. All but one participant reported using a laptop or computer, and the participant who did not was excluded from analyses along with those who did not complete the study (11 participants did not complete the study; 12 were excluded in total). The mean age of the final sample (*n* = 91; 47 female and 44 male) was 38.76 years (standard deviation (SD) = 12.99, range = 19–70). The mean BMI (calculated from self‐reported weight and height) was 27.99 (SD = 7.51, range = 16.26–54.29). The majority of participants were Caucasian (81.3%). The sample were generally well educated with the majority having had some experience of college or a bachelor's degree (83.6%), and the majority (58.3%) earned below $40,000. The study was approved by the authors' institutional ethics board (as were Studies 2 and 3). Participants received a small financial remuneration (50 cents) for their time.

#### Stimuli

The stimuli consisted of photographs of Caucasian men and women with varying BMIs (calculated from measured weight [kg]/height^2^ [m]). The photographed individuals were students and staff recruited from the University of Birmingham (men) and the University of Liverpool (women) in the UK. The models were stood next to a standard door frame, wearing normally fitting short‐sleeved t‐shirts and full‐length trousers or leggings. No models had particularly muscular builds (determined by fat mass percentage; the men had body fat >8%, and the women had a body fat percentage >21%), and the central section of each model's face was obscured. In order to select standardized images of men and women for use in these three studies, a pilot study was conducted in which 40 US participants rated appearance‐related dimensions of the photographs, such as attractiveness, posture, how muscular the target appeared and tightness of clothing. Twenty‐one photographs of male models and 21 photographs of female models were selected (with equal numbers of models in the normal weight range [BMI = 18.5–24.9], overweight range [BMI = 25.0–29.9] and obese range [BMI = 30–39.9]) that scored similarly on these dimensions. All selected models were aged 18–40 years (see Supporting Information for example images and for BMI information of the selected models).

#### Procedure

The study was advertised as being about how people make judgements about others. Participants provided digital informed consent and were given World Health Organization BMI guidelines for underweight (<18.5), normal weight (18.5–24.9), overweight (25.0–29.9), obese (30–39.9) and severely obese (>40) weight statuses. Participants viewed each of the 42 photographs consecutively on separate pages in a random order and were asked to estimate the weight category of each photographed person. Participants then provided demographic information (sex, age, ethnicity, height, weight, education and income) and were debriefed. Participants were allocated up to 60 min to complete the survey.

### Analysis

The majority of the time that participants were inaccurate, they were underestimating, rather than overestimating, the weight status of the models (Table 1). Thus, the main analysis focused on underestimation of weight status. Underestimation was characterized by calculating a score out of seven to represent the number of times participants underestimated the weight status of models from each weight category (normal weight, overweight and obese men and women). A 2 × 3 repeated measures analysis of variance was planned with sex (male or female) and weight status (normal weight, overweight or obese) of model as within subject factors and frequency of underestimation as the dependent variable. If a significant interaction was found between model sex and weight status, Bonferroni‐corrected *t*‐tests examining the difference in underestimation between male and female models with normal weight, overweight and obese BMIs separately were planned. The effect of participant demographics on underestimation was also examined; participant demographic variables that were associated with frequency of underestimation (at a conservative level of *p* ≤ 0.20) were controlled for in the primary analysis in order to rule out any potential confounds. All data were significantly skewed according to the Kolmogorov–Smirnov test of normality (*p*s < 0.001), and the data were log transformed (as was the case in Studies 2 and 3). Inferential statistics (including effect sizes) were conducted on log‐transformed data. Means that are reported are based on the non‐transformed data for ease of interpretation.

**Table 1:** Percentage of underestimation, accuracy and overestimation of male and female photographs in Study 1

Sex	Weight status	Underestimated (%)	Accurate (%)	Overestimated (%)
Male	Normal weight	32	67	1
	Overweight	79	21	0
	Obese	90	10	0
Female	Normal weight	14	79	7
	Overweight	30	60	10
	Obese	62	35	3

Participants judged seven photographs of men and seven photographs of women from the three weight status categories.

### Results

#### Underestimation

The frequency of underestimation by weight status and sex is presented in Table 1. There was a significant main effect of model sex (*F*(1, 90) = 303.88, *p* < 0.001, *η*_*p*_^2^ = 0.77), participants underestimated the weight status of male models (67%) more frequently than female models (36%). There was also a significant effect of model weight status (*F*(2, 180) = 303.13, *p* < 0.001, *η*_*p*_^2^ = 0.77), whereby the weight of obese models was more frequently underestimated (76%) than overweight (54%) (*p* < 0.001, *d* = 2.64) or normal weight models (23%) (*p* < 0.001, *d* = 3.0). The weight status of overweight models was also underestimated significantly more frequently than normal weight models (*p* < 0.001, *d* = 2.76). Finally, there was a significant interaction between model sex and model weight status (*F*(2, 180) = 48.86, *p* < 0.001, *η*_*p*_^2^ = 0.35). The weight status of male models was consistently underestimated more than female models, and the interaction was driven by a particularly large sex difference in underestimation within the overweight range (see Table 2 for Bonferroni‐corrected *t*‐tests, means and SDs). The effect of participant demographics on underestimation were also examined. Only level of education (*p* = 0.068) was marginally associated with frequency of underestimation. Sex (*p* = 0.580), age (*p* = 0.433), BMI (*p* = 0.449), income (*p* = 0.931) and ethnicity (this was operationalized as White or not due to the small proportions of non‐White participants) (*p* = 0.622) were not associated with underestimation. When level of education was included as a covariate in the 2 × 3 analysis of variance discussed previously, the pattern of results was the same. The main effects of sex (*F*(1, 89) = 7.36, *p* = 0.008, *η*_*p*_^2^ = 0.08), weight status (*F*(2, 178) = 23.28, *p* < 0.001, *η*_*p*_^2^ = 0.21) and the interaction between sex and weight status (*F*(2, 178) = 3.60, *p* = 0.029, *η*_*p*_^2^ = 0.04) remained significant.

**Table 2:** Means (SD) and *t*‐test results for underestimation scores for male and female photographs in Study 1

	Female models	Male models	*t*‐test result
Normal weight	1.01 (0.67)	2.23 (1.68)	*t*(90) = 7.35, *p* < 0.001, *d* = 0.95
Overweight	2.11 (1.81)	5.52 (1.50)	*t*(90) = 17.59, *p* < 0.001, *d* = 2.04
Obese	4.35 (1.93)	6.29 (1.03)	*t*(90) = 11.08, *p* < 0.001, *d* = 1.25

Means refer to the average number of models' weight status, which was underestimated (as participants estimated the weight status of seven male and seven female models, the mean is out of 7).

### Discussion

Participants frequently underestimated the weight status of both men and women with overweight and obesity. The frequency of underestimation was higher when the models were male, as opposed to female. Moreover, this sex difference was particularly pronounced when the models were overweight.

## Study 2

Body size ‘norm comparison’ processes may be responsible for the visual underestimation of overweight and obesity evidenced in Study 1 12. A prototype explanation 9 suggests that the body size a person perceives as being ‘average’ affects how weight status is judged, whereby body sizes are judged in comparison with a person's perception of the ‘average’ body. Based on visual categorization theory 11, there is a range of body sizes categorized as being ‘normal’, and how a body compares with the largest body within the ‘normal range’ of body sizes is critical. Study 2 examined whether either of these processes predicts when the weight status of men and women with overweight is visually underestimated. As underestimation was common in the overweight range in Study 1, Study 2 focused on the overweight BMI range.

### Method

#### Participants

One hundred and two US participants were recruited through Amazon Turk; the same criteria were used as in Study 1 in order to ensure quality of data. Participants from Study 1 were ineligible to participate, and the Unique Turker function was used to ensure that participants from Study 1 could not participate in Study 2. Participants were asked to complete the survey on a computer or laptop, and all participants reported complying with this rule. Participants were excluded from final analyses if they did not complete the study (23 participants started but did not complete the study). The final sample of 79 participants (41 female and 38 male) had a mean age of 37.41 years (SD = 12.66, range = 19–67), and their mean BMI (calculated from self‐reported weight and height) was 26.06 (SD = 5.87, range = 16.55–45.56). The majority of participants were Caucasian (83.5%). The sample were generally well educated with the majority having had some experience of college or a bachelor's degree (78.5%), and the majority (60.8%) earned below $40,000. Participants received remuneration (50 cents) for their time.

#### Procedure

**Figure 1:**
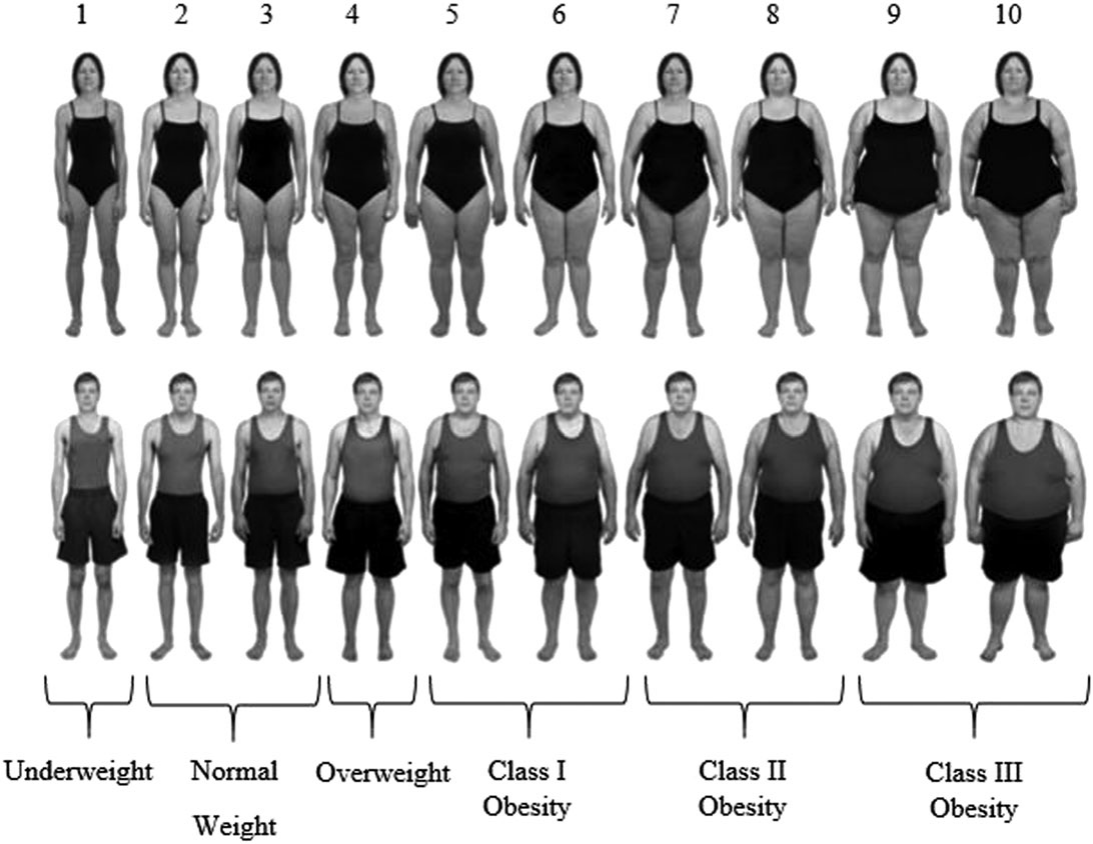
Female and male body size guide images 30 with rating scale 1, 2, 3, 4, 5, 6, 7, 8, 9, 10 and weight status (according to objectively measured body mass index) below.

The study was advertised as being about how people make judgements about people that they do not know. Participants gave digital consent and were given the same BMI guidance as in Study 1. They then viewed 14 photographs, featuring the same overweight models as in Study 1, in a random order on separate pages and were asked to estimate the weight status of each model. To measure body size norms, participants were next shown male and female body size guides (BSGs) 29; validated rating scales consisting of photographs of 10 standardized human bodies of increasing BMI, ranging from underweight to class III obesity (Figure 1). Participants were asked to select the body size (for men and women separately) that they thought ‘best represented an average size’ and were asked to select all of the body sizes they believed ‘looked normal in size’. The order in which participants completed these measures was randomized. Participants then provided demographic information (sex, age, ethnicity, height, weight, education and income) and were debriefed. Participants were allocated up to 60 min to complete the survey.

### Analysis

Underestimation was characterized by the number of photographs (out of seven), for which a participant underestimated the weight status of the model. Participants were also given scores (1 being the slimmest image and 10 being the largest) for the male and female body sizes they selected as being average (average) and the slimmest and largest body sizes they selected as falling within a normal range (lower and upper norm boundary) using the BSGs. As it is conceivable that the number of body sizes perceived as being normal could influence judgements, the width of the norm range (number of bodies selected as being ‘normal’) was also computed (norm width). To examine differences in judgements made about male and female models, sex discrepancy scores were then calculated by subtracting the male score from the female equivalent for each of the aforementioned measures.

Stepwise regression analyses were planned to compare the different norm judgments in terms of the extent to which they predicted underestimation of weight status, as this regression model automatically selects the strongest predictors and removes non‐significant predictors. To examine which norm measures best predicted underestimation of weight status for men and women separately, two stepwise regression analyses were planned with the upper and lower norm boundary, average and norm width as predictor variables and frequency of underestimation as the outcome variable. In order to examine whether a discrepancy in what was perceived as being normal for men vs. women predicted why male overweight was underestimated more frequently than female overweight, a further stepwise regression model was conducted. In this model, the predictor variables were the sex discrepancy (difference in score between male vs. female models) in the upper and lower norm boundary, norm width and average body size, and sex discrepancy in underestimation of weight status was the outcome variable. Finally, for each stepwise regression model, demographic factors that were associated with underestimation (as in Study 1; at a conservative level of *p* ≤ 0.20) were controlled for.

### Results

#### Underestimation and norm judgements

In line with Study 1, participants underestimated the weight status of significantly more male models (84%) than female models (36%) (*t*(78) = 17.18, *p* < 0.001, *d* = 2.32). Participants believed that an average male body size (*M* = 4.28, SD = 1.15) was larger than the average female body size (*M* = 3.70, SD = 1.18; *t*(78) = 5.45, *p* < 0.001, *d* = 0.60). Similarly, participants selected larger lower and upper norm boundaries for male models (lower *M* = 2.91, SD = 1.07; upper *M* = 4.80, SD = 1.37) than female models (lower *M* = 2.39, SD = 1.11; upper *M* = 4.24, SD = 1.60; lower = *t*(78) = 5.84, *p* < 0.001, *d* = 0.54; upper = *t*(78) = 5.16, *p* < 0.001, *d* = 0.36). The width of the normal range was similar for male (*M* = 2.86, SD = 1.83) and female models (*M* = 2.85, SD = 1.97; *t*(78) = 0.35, *p* = 0.726, *d* < 0.01).

#### Male underestimation

The regression model examining male underestimation was statistically significant (*F*(1, 78) = 14.46, *p* < 0.001, Δ *R* ^2^ = 0.15), and the upper norm boundary was identified as a significant predictor of underestimation (*B* = 0.259, SE = 0.07, *β* = 0.398, *t* = 3.80, *p* < 0.001). The lower norm boundary, average and norm width (all *p*s > 0.05) did not predict underestimation (Table 3). For each one unit increase in the upper norm boundary, frequency of underestimation increased by 4%. There was no evidence of significant multicollinearity (variance inflation factor [VIF] < 3). In order to be sure that demographic factors were not influencing underestimation, further analyses were conducted examining whether any of the main results differed when controlling for participant demographic variables that were associated with underestimation at *p* ≤ 0.20. For male underestimation, neither sex (*p* = 0.273), age (*p* = 0.543), ethnicity (White or not) (*p* = 0.680), education level (*p* = 0.980), income (*p* = 0.905) or BMI (*p* = 0.895) were associated with underestimation at *p* ≤ 0.20, so no further analyses were conducted.

**Table 3:** Standardized beta, *t* values and *p* values for non‐significant predictors in the stepwise regression models for male and female underestimation and the discrepancy in underestimation in Study 2

	Upper norm boundary	Lower norm boundary	Average	Norm width
Underestimation of male overweight	*β* = 0.259, *t* = 3.80, *p* < 0.001	*β* = 0.095, *t* = 0.90, *p* = 0.369	*β* = −0.040, *t* = 0.36 *p* = 0.723	*β* = −0.141, *t* = 0.90, *p* = 0.374
Underestimation of female overweight	*β* = 0.417, *t* = 4.25, *p* < 0.001	*β* = 0.186, *t* = 1.84, *p* = 0.069	*β* = 0.213, *t* = 1.93, *p* = 0.057	*β* = −0.297, *t* = 1.92 *p* = 0.059
Discrepancy between underestimation of male and female overweight	*β* = 0.398, *t* = 2.94, *p* = 0.004	*β* = 0.154, *t* = 1.29, *p* = 0.200	*β* = 0.099, *t* = 0.87 *p* = 0.386	*132* = −0.167, *t* = 1.22, *p* = 0.225

#### Female underestimation

The regression model examining female underestimation was statistically significant (*F*(1, 78) = 18.05, *p* < 0.001, Δ *R* ^2^ = 0.18). The upper norm boundary was identified as a significant predictor of underestimation (*B* = 0.417, SE = 0.10, *β* = .436, *t* = 4.25, *p* < 0.001). The lower norm boundary, average and norm width (all *p*s > 0.05) did not predict underestimation (Table 3). For each one unit increase in the upper norm boundary, frequency of underestimation increased by 6%. There was no evidence of significant multicollinearity (VIF < 3). Education (*p* = 0.043), BMI, (*p* = 0.039) and income (*p* = 0.137) were associated with underestimation at *p* ≤ 0.20, whereas sex (*p* = 0.491), age (*p* = 0.401) and ethnicity (White or not) (*p* = 0.576) were not. After controlling for BMI, education and income, the upper norm boundary was still a significant predictor of underestimation (*B* = 0.417, SE = 0.10, *β* = .436, *t* = 4.25, *p* < 0.001).

#### Sex discrepancy in underestimation

The regression model examining sex discrepancy in underestimation was statistically significant (*F*(1, 78) = 8.65, *p* = 0.004; Δ *R* ^2^ = 0.09). The upper norm boundary discrepancy was identified as a significant predictor of underestimation discrepancy (*B* = 0.398, SE = .14, *β* = .318, *t* = 2.94, *p* = 0.004). The lower norm boundary discrepancy, average discrepancy and norm width discrepancy (all *p*s > 0.05) did not predict underestimation discrepancy (Table 3). For every one unit difference between male and female upper norm boundaries, the tendency for male overweight to be underestimated more than female overweight increased by 5%. There was no evidence of significant multicollinearity (VIF < 3). Sex (*p* = 0.173), education (*p* = 0.035), income (*p* = 0.159) and BMI (*p* = 0.023) were associated with the sex discrepancy in underestimation at *p* ≤ 0.20, whereas age (*p* = 0.216) and ethnicity (White or not) (*p* = 0.995) were not. After controlling for sex, education, income and BMI, the discrepancy in upper bounds was still a significant predictor of the discrepancy in underestimation (*B* = 0.398, SE = 0.14, *β* = 0.318, *t* = 2.94, *p* = 0.004).

### Discussion

In line with Study 1, the weight status of men and women with overweight was frequently underestimated. The results of Study 2 supported a categorization theory of body norms and weight status underestimation 11. The results suggest that there is a range of body sizes that are perceived as being normal in size, and when a target body is bigger than the largest body size in this ‘norm range’ (the ‘upper norm boundary’), underestimation of weight status is more likely to occur. Furthermore, sex differences in this ‘upper norm boundary’ was associated with male overweight being more frequently underestimated than female overweight; the largest body size perceived as being ‘normal’ was bigger for men than women.

## Study 3

The aim of Study 3 was to directly examine the hypothesis that exposure to obesity results in an upwards shift in the range of body sizes that are perceived as being ‘normal’, resulting in visual underestimation of weight status. Based on the findings of Study 2, it was expected that exposure to obesity would result in an upwards shift in the largest body size perceived as being normal (the upper norm boundary), leading to increased underestimation of weight status. In Study 3, some of the potential limitations associated with the stimuli used in Studies 1 and 2 were addressed. The images in the first two studies were not fully standardized (e.g. participant clothing varied between stimuli). Although it is unlikely that these factors would have affected the pattern of results observed, ideally, stimuli should be as standardized as is possible in terms of both clothing and colour 31. As such, stimuli in Study 3 were presented in greyscale, and standardized images were taken from the BSG; the validated body image scale used in Study 2 29.

### Method

#### Participants

Because Study 3 involved an experimental manipulation, a larger sample size was recruited, and the study was powered to detect medium‐sized between‐subjects effects 16. Three hundred and twenty‐four US participants were recruited via Amazon Mechanical Turk, and Unique Turker was used to ensure that participants who took part in Studies 1 and 2 did not take part in Study 3. The same criteria was used as in Studies 1 and 2 in order to ensure quality of data. Participants who failed to complete study instructions (34 participants) were excluded from analyses. Participants were asked to complete the survey on a computer or laptop and received remuneration (50 cents) for their time. The final sample of 290 participants (174 female and 116 male) had a mean age of 35.55 years (SD = 12.40, range = 18–77) and a mean BMI of 28.66 (SD = 9.59, range = 14.68–74.45). The majority of participants were Caucasian (76.9%), had some experience of college or a bachelor's degree (72.7%) and earned below $40,000 (60%).

#### Procedure

Participants were told that the aim of the study was to examine how personality impacted judgements about others (cover story). After providing consent, participants provided demographics (sex, age, ethnicity, height, weight, education and income) and completed personality questions (e.g. ‘I am an outgoing person’) to distract from the study aims. Participants either made ratings about images of male or female models (between‐subjects). In the exposure phase of the experiment, participants were exposed to 10 images of BSGs (Figure 1) with either normal weight (BMI = 18.5–24.9) or obese (BMI = 35–39.9) BMIs (between‐subjects) on consecutive pages and were asked to make one non‐weight‐related judgements about each image (e.g. ‘he/she looks like he/she would be kind’). This procedure allowed us to visually expose participants to different body sizes in a way that corroborated the study cover story. Participants then completed the norm judgement questions as in Study 2 (BSGs 29). Finally, participants were asked to estimate the weight status of an overweight male or female BSG (the sex of the overweight BSG being evaluated was the same as the sex of BSGs participants were exposed to). The presentation of the norm and weight judgement tasks was counterbalanced. Participants were then asked to guess the aims (none of the participants accurately guessed the aims of the study) and were debriefed. Participants were allocated up to 60 min to complete the survey.

### Analysis

Separate analyses for judgements about women and men were conducted. A series of *t*‐tests were planned to examine whether exposure condition (normal weight or obese) impacted judgements about norms (upper norm boundary, lower norm boundary, average and norm width), and chi‐squares were used to examine whether exposure condition impacted frequency of underestimation. Next, binary logistic regression analyses were planned to examine whether any of the norm judgements that differed significantly between exposure conditions were independently associated with underestimation. If this was the case, PROCESS mediation analyses 30 were planned in order to examine whether the effect of exposure to obesity on underestimation of weight status was mediated by alteration to body size norm measures. In order to examine whether results were consistent, any demographic factors that were associated with underestimation (at a conservative level of *p* ≤ 0.20) were controlled for in the mediation analyses.

### Results

#### The effect of exposure on judgements about women

**Figure 2:**
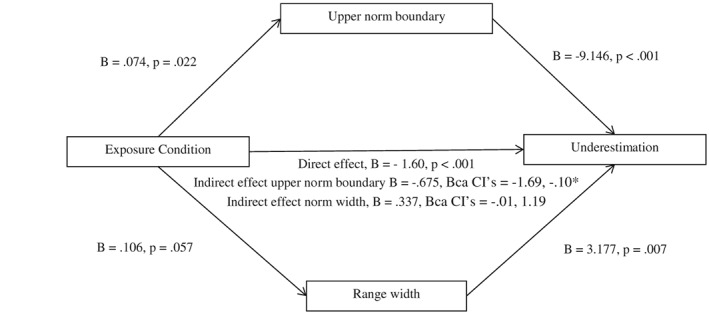
Model of exposure condition as a predictor of underestimation mediated by upper norm boundary and range width in women. The confidence interval (CI) for the indirect effect is a bias‐corrected and accelerated (BCa) bootstrapped CI based on 1,000 samples.*Indicates statistical significance.

Participants who were exposed to women with obesity later underestimated the weight status of the woman with overweight (43%) significantly more than participants who were exposed to normal weight women (13%). Furthermore, participants exposed to women with obesity chose a larger body size as being the largest body that fell within the ‘normal’ range (upper norm boundary) than participants exposed to normal weight women. Participants in the obese exposure condition also selected a larger ‘average’ body size and had a borderline significantly larger norm width than participants in the normal weight exposure condition. Lower bound judgements did not differ between exposure conditions (see Table 4 for chi‐squared and *t*‐test results). In the binary logistic regression model, upper norm boundary (*B* = −9.391, SE = 2.50, *p* < 0.001) and norm width (*B* = −3.084, SE = 1.17, *p* = 0.008) were significantly associated with underestimation, whereby a larger upper norm boundary and norm width predicted underestimation. The average norm was not associated with underestimation (*B* = 0.576, SE = 1.78, *p* = 0.746). In the parallel PROCESS mediation model, the upper norm boundary significantly mediated the relationship between condition and underestimation (*B* = −0.675, bias‐corrected and accelerated confidence intervals (BCa CIs) = −1.69, −0.10), whereas norm width did not (*B* = 0.337, BCa CIs = −0.01, 1.19; Figure 2). Participant age (*p* = 0.008), education (*p* < 0.001) and BMI (*p* = 0.025) were all associated with underestimation at a level of *p* ≤ 0.20, whereas sex (*p* = 0.550), income (*p* = 0.865) and ethnicity (White or not) (*p* = 0.582) were not. When age, education and BMI were included as covariates in the parallel mediation model described previously, the pattern of results did not change. The upper norm boundary still mediated the relationship between condition and underestimation when age (*B* = −0.626, BCa CIs = −1.54, −0.03), education (*B* = −0.721, BCa CIs = −1.94, −0.06) and BMI (*B* = −0.645, BCa CIs = −1.64, −0.08) were included as covariates.

**Table 4:** The effect of experimental exposure condition on norm judgements and underestimation in Study 3

	Normal weight exposure	Obese exposure	Test results
Female (*N* = 142)	(*N* = 68)	(*N* = 74)	
Upper norm boundary	4.66 (2.28)	5.28 (1.97)	*t*(140) = −2.31, *p* = 0.022, *d* = 0.37
Lower norm boundary	2.18 (1.01)	2.31 (1.38)	*t*(140) = −0.08, *p* = 0.935, *d* = 0.01
Average	3.60 (1.07)	4.32 (1.29)	*t*(140) = −3.36, *p* = 0.001, *d* = 0.52
Norm width	3.47 (2.72)	3.91 (2.17)	*t*(140) = −1.92, *p* = 0.057, *d* = 0.33
Underestimation of weight status	9 (13%)	32 (43%)	*χ* ^*2*^(1, *N* = 142) = 15.54, *p* < 0.001, *V* = 0.33
Male (*N* = 148)	(*N* = 75)	(*N* = 73)	
Upper norm boundary	4.56 (1.50)	5.52 (1.98)	*t*(146) = −3.27, *p* = 0.001, *d* = 0.50
Lower norm boundary	2.67 (0.88)	2.74 (1.01)	*t*(146) = −0.048, *p* = 0.962, *d* = 0.01
Average	4.18 (1.10)	4.78 (1.19)	*t*(146) = −3.20, *p* = 0.002, *d* = 0.50
Norm width	2.88 (1.82)	3.78 (2.42)	*t*(146) = −2.12, *p* = 0.036, *d* = 0.35
Underestimation of weight status	62 (83%)	67 (92%)	*χ* ^*2*^(1, *N* = 148) = 2.75, *p* = 0.097, *V* = 0.14

For upper norm boundary, lower norm boundary and average, values refer to body sizes selected using body size guide scales and are *M* (SD). Norm width refers to number of body sizes selected using body size guide scales are *M* (SD). Underestimated refers to number of participants underestimating the weight status of the overweight model (frequency [%]).

#### The effect of exposure on judgements about men

**Figure 3:**
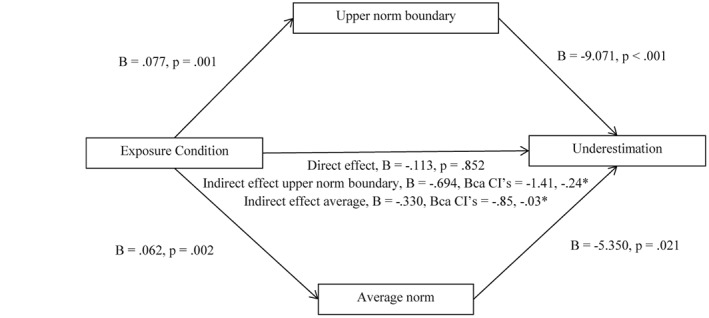
Model of exposure condition as a predictor of underestimation mediated by upper norm boundary and average norm in men. The confidence interval (CI) for the indirect effect is a bias‐corrected and accelerated (BCa) bootstrapped CI based on 1,000 samples. *Indicates statistical significance.

Underestimation tended to be more common after being exposed to men with obesity (92%), as opposed to normal weight men (83%). This did not reach statistical significance (*p* = 0.097), but this may be due to the high prevalence of underestimation in both conditions. Participants exposed to obesity selected a larger body as the upper norm boundary and a larger body size as being average, as well as selecting a wider norm width. Exposure condition had no impact on the lower norm boundary (Table 4). In the binary logistic regression model, upper norm boundary (*B* = −12.266, SE = 4.01, *p* = 0.002) and average norm (*B* = −5.066, SE = .226, *p* = 0.025) were significantly associated with underestimation, whereby a larger upper norm boundary and average predicted underestimation. Norm width was not associated with underestimation (*B* = 1.940, SE = 1.86, *p* = 0.298). In the parallel PROCESS mediation model, a significant indirect effect of condition on underestimation through the upper norm boundary was observed (*B* = −0.694, BCa CIs = −1.41, −0.24), as well as through the average norm (*B* = −0.330, BCa CIs = −0.85, −0.03; Figure 3). Participant ethnicity (White or not) (*p* = 0.011) and age (*p* = 0.041) were associated with underestimation at a level of *p* ≤ 0.20, whereas sex (*p* = 0.976), income (*p* = 0.438) education (*p* = 0.267) and BMI (*p* = 0.656) were not. The indirect effects of both the upper norm boundary (ethnicity = *B* = −0.664, BCa CIs = −1.36, −0.23; age = *B* = −0.579, BCa CIs = −1.20, −0.17) and the average (ethnicity = *B* = −0.307, BCa CIs = −0.82, −0.01; age = *B* = −0.300, BCa CIs = −0.78, −0.02) remained significant when ethnicity and age were included as covariates in the parallel mediation model.

### Discussion

Visual exposure to obesity shifted the range of body sizes perceived as being ‘normal’ upwards, which acted as a mediator in explaining the effect of exposure to obesity on visual underestimation of overweight. Norms regarding what an average weight looked like also mediated the relationship between exposure to obesity and underestimation when judging the overweight status of men but not women.

## General Discussion

The present studies suggest that overweight and obesity are under‐detected visually, which may be caused by exposure to larger body sizes having changed the range of body sizes, which are perceptually judged as being ‘normal’. The present findings support a ‘norm comparison’ theory of the underestimation of weight status 3, 13. This theory suggests that bodies that are perceived as being outside of the range of body sizes that are considered to be normal will be judged as being overweight. The increased prevalence of obesity is likely to have resulted in heavier body sizes being perceived as being ‘normal’. These findings could explain why visual underestimation of obesity is more common in countries with a higher obesity prevalence 32 and why individuals with overweight peers are more likely to underestimate their own weight status 15.

Previous work has found that male overweight and obesity is visually underestimated 2, 6, 32, and here, this was also found to be the case for female overweight and obesity. However, the weight status of men with overweight was more likely to be underestimated than that of women. Media influence could be partly responsible for the sex discrepancy in underestimation as female models and actresses are more likely to be slender than male models 33, 34, and there is a persuasive western ‘thin ideal’, whereby thinness is valued more positively and presented more frequently for women than men 25. These factors are therefore likely to result in thinner body sizes appearing more normal for women than men, as was the case in Study 2. In line with this, there is evidence that women are more likely to overestimate their weight status than men 35, 36. Future research should examine the separate and combined effects of exposure to heavier and slimmer bodies in the media and in everyday life on body size norms and perception of weight.

The implications and applied relevance of the present studies now require further attention. Some researchers suggest that a failure to identify overweight and obesity in others could be a barrier to weight loss, as family members 37 and clinicians 38 could be important agents of change in terms of promoting healthier behaviours. Parents who underestimate child overweight are less likely to be concerned about their child's weight 39 and are less likely to attempt a weight loss intervention 40, 41. Furthermore, general practitioners are less likely to discuss weight loss interventions with patients when they underestimated the patients' weight 6. These studies are suggestive of a need for more accurate recognition of overweight and obesity. This could be achieved by training parents and HCPs to recognize body size norms, which represent a healthier weight.

Conversely, an emerging literature suggests that underestimation of overweight may not be a barrier to weight loss, as self‐identification of overweight has been shown to be associated with a number of adverse outcomes, including greater body dissatisfaction 42, depression 43 and weight gain 44, 45. These findings are consistent with a broader literature on obesity and body satisfaction, which shows that many individuals with obesity report higher body dissatisfaction 46, 47, which can impact on self‐esteem and depression 46. These findings are likely to be at least in part due to the stigma of obesity 48, 49, 50, which could make identifying as being overweight or obese unpleasant. One solution to this would be to ensure that weight information is relayed to patients in a sensitive and non‐stigmatizing way and to build stigma reduction techniques into future weight loss interventions. Furthermore, different strategies for reducing the potentially negative effects that self‐perceived overweight can have on body satisfaction, weight‐related behaviours and weight gain may warrant investigation.

A limitation of the present studies was that the sample was predominantly Caucasian (81% average across the three studies). Similarly, the models used as stimuli in the studies were Caucasian. Some studies suggest that identification of overweight 51 and body norms 52 can be affected by ethnicity, so further work in more diverse samples would now be valuable. It may also be the case that overweight and obesity are more easily detected in person than when using photograph stimuli, and this may have resulted in poorer identification of weight status in the present studies. However, it should be noted that there is convincing evidence of widespread under‐detection of overweight and obesity when judgements are made in person 53, 54. As all three studies were conducted online, a further limitation could be reduced control over participant responses. However, we used a number of procedures to limit this concern; we sampled only reliable participants from MTurk (determined by their previous approval ratings) and included attention checks to detect whether participants were completing our studies as intended. Finally, there may be some limitations associated with the photographic stimuli used in Studies 1 and 2. Although the male and female sets were closely matched in terms of the BMI and appearance of the models, the male and female photograph sets used in Studies 1 and 2 are not perfectly matched (e.g. in terms of the clothing worn by models). However, the same pattern of results observed in Studies 1 and 2 was observed in Study 3 where the images used were standardized.

Overweight and obesity are under‐detected visually. The visual under‐detection of overweight and obesity may be in part caused by exposure to obesity changing the range of body sizes that are perceptually judged as being ‘normal’.

## Conflict of Interest Statement

The authors declared that they had no conflicts of interest with respect to their authorship or the publication of this article.

## Funding

This research received no external funding. E. R.'s salary is supported by the Medical Research Council and the Economic and Social Research Council. E. R. has also received research funding from the Wellcome Trust, the National Institute of Health Research, the American Beverage Association and Unilever.

## Supporting information


**Table S1.** Mean and range of BMI for male and female models from each weight status group
**Figure S1.** Sample photographs from overweight range
