# Supplementary material for: Visual body size norms and the under‐detection of overweight and obesity
Source: Obes Sci Pract. 2017 Dec 21;4(1):29–40. doi: 10.1002/osp4.143 (PMC5818735; doi:10.1002/osp4.143)
Supplement: Supplementary file 1 — Table S1. Mean and range of BMI for male and female models from each weight status group Figure S1. Sample photographs from overweight range [file OSP4-4-29-s001.docx]

**Supplement For:**

**Visual Body Size Norms and the Under-Detection of Overweight and Obesity**

**Supplemental Table 1.** Mean and range of BMI for male and female models from each weight status group

|  | Male | Females |
| --- | --- | --- |
| Normal weight | 21.33 (18.68-23.84) | 21.41 (18.75-23.63) |
| Overweight | 27.62 (25.70-29.44) | 27.64 (25.81-29.38) |
| Obese | 31.79 (30.49-34.42) | 31.71 (30.11-34.26) |


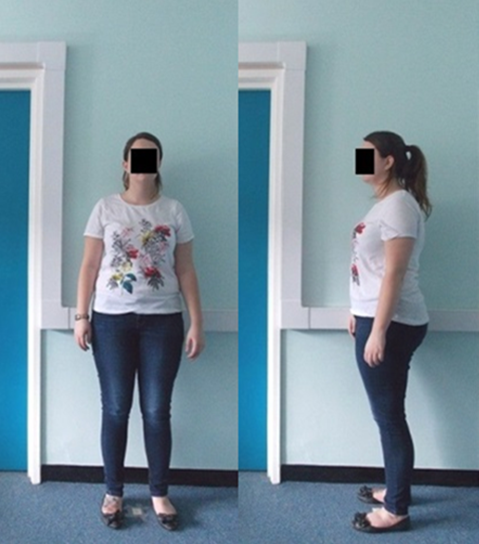

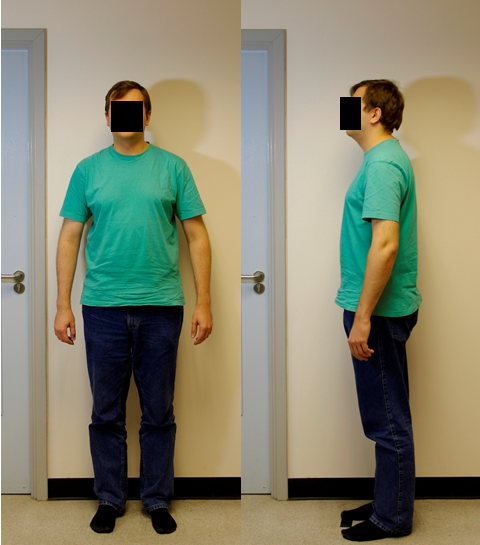
**Supplemental Figure 1** Sample photographs from overweight range
